# Arterial Structure and Function in Ambulatory Adolescents with Cerebral Palsy Are Not Different from Healthy Controls

**DOI:** 10.1155/2012/168209

**Published:** 2012-06-10

**Authors:** Audra A. Martin, Lisa M. Cotie, Brian W. Timmons, Jan Willem Gorter, Maureen J. MacDonald

**Affiliations:** ^1^Department of Kinesiology, McMaster University, 1280 Main Street West, Hamilton, ON, Canada L8S 4K1; ^2^Child Health & Exercise Medicine Program, McMaster University, 565 Sanatorium Road, Hamilton, ON, Canada L8N 3Z5; ^3^CanChild Centre for Childhood Disability Research, McMaster University, 1400 Main Street West, Hamilton, ON, Canada L8S 1C7

## Abstract

Physical inactivity in youth with cerebral palsy (CP) places them at increased risk of developing cardiovascular disease. The current study assessed indices of arterial health in adolescents with CP, classified as levels I-II of the Gross Motor Function Classification System (GMFCS) (*n* = 11, age 13.2 ± 2.1 yr), in comparison to age- and sex-matched controls (*n* = 11, age 12.4 ± 2.3 yr). Groups were similar in anthropometric measurements, resting blood pressures, and heart rates. There were no group differences in brachial flow-mediated dilation (11.1 ± 7.8 versus 6.1 ± 3.6), carotid intima-media thickness (0.42 ± 0.04 versus 0.41 ± 0.03 mm), and distensibility (0.008 ± 0.002 versus 0.008 ± 0.002 mmHg) or central (4.3 ± 0.6 versus 4.1 ± 0.9 m/s) and peripheral pulse wave velocity (7.1 ± 1.7 versus 7.6 ± 1.1 m/s); CP versus healthy controls, respectively. Vigorous intensity physical activity (PA) was lower in the CP group (CP: 38 ± 80 min versus controls: 196 ± 174 min); groups were similar in light and moderate intensity PA levels. Arterial health of ambulatory youth with CP is not different from a control group despite lower vigorous PA levels. Similar studies need to examine individuals with more pronounced mobility limitations (GMFCS level III–V).

## 1. Introduction

Cerebral Palsy (CP) is defined as a disorder of posture and movement due to a nonprogressive disturbance in the developing fetal or infant brain [1]. CP manifests as limitations in gross motor capacity [2], affecting performance in daily mobility over a lifespan [3]. Youth with CP are less physically active than their typically developing peers [4, 5] and show an inverse relationship between functional limitations and social participation [6]. Physical inactivity in youth places them at a greater risk of developing a variety of secondary health complications [7] and is also a major controllable risk factor for cardiovascular disease (CVD) [8].

It has been suggested that one mechanism by which physical activity (PA) exerts its protective effect on cardiovascular health is through positive effects on the endothelium [9], a single layer of cells responsible for the vasodilator response to increased conduit artery flow. A strong relationship between low levels of PA and endothelial dysfunction has been well documented in children [10], potentially predisposing youth with CP to an increased risk of endothelial dysfunction. Endothelial dysfunction is considered an early and integral manifestation of atherosclerotic disease, which can be evident in the first decade of life [11]. Endothelial dysfunction is an indicator of preclinical vascular disease and for youth with CP may act as a marker of early changes in vessel function, indicative of future atherosclerotic risk [12].

Pulse wave velocity (PWV) is a sensitive marker of arterial wall stiffness and subsequent marker of cardiovascular risk [13]. In children, PWV is positively correlated with body mass index (BMI), waist circumference (WC), and percentage body fat and negatively correlated with cardiorespiratory fitness and levels of PA [14]. Carotid artery distensibility and carotid artery intima-media thickness (_c_IMT) are two additional indices of arterial health and their role in the development of CVD is widely accepted [15].

Strong relationships between cardiovascular risk factors identified in childhood and adolescence and the progression of atherosclerosis in adulthood are emerging [16]. Consistent, positive effects of habitual PA on vessel health have been demonstrated [14, 17]. Measuring indices of arterial stiffness and endothelial function are therefore important in this young, clinical population in order to identify changes in vascular health at the earliest stage possible.

To our knowledge, there is no study published assessing vessel health in general or its association with levels of habitual PA in youth with CP. Given the fact that children aged 5 to 7 years with CP have lower PA levels than typically developing peers [4, 5] we hypothesize that even the most functional adolescents with CP (GMFCS levels I-II) may have decreased levels of PA and altered arterial function and structure compared to an age- and sex-matched control sample.

## 2. Methods

### 2.1. Participants

Twenty-two adolescents (9–16 yrs) were recruited; of which, 11 individuals with CP (8 boys; mean ± SD age of 13.2 ± 2.1 yr) were recruited from the Spasticity Clinic and Teen Transition Clinic at McMaster Children's Hospital, Hamilton, Ontario, Canada. Inclusion criteria for the CP group included a classification of either a level I or II (GMFCS-Expanded & Revised) [18] indicating that all subjects with CP were ambulatory without use of mobility devices (level I *n* = 7, level II *n* = 4). Subjects were chronological age- and sex-matched to a healthy control group with a mean age of 12.4 ± 2.3 yr. Control subjects were healthy, with no known cardiovascular or metabolic conditions and studied without specific exclusion criteria. Experimental procedures were explained to participants and their guardians prior to obtaining written and verbal informed consent/assent from the parent/guardian and participant, respectively. Approval from the Hamilton Health Sciences and McMaster University Faculty of Health Sciences Research Ethics Board was obtained for the study.

### 2.2. Study Design

This study employed a cross-sectional design to characterize the differences in specific measures of vascular structure and function between children with CP and healthy controls. All measures were noninvasive and took place in a quiet, temperature-controlled room (23° ± 1°C) with the participant in a supine position. All subjects were instructed to abstain from vigorous PA 24 hours pre- and were tested 4 hours postprandialy [19].

#### 2.2.1. Anthropometric Measurements

 Sitting and standing height (cm) were measured to the nearest mm without shoes and in light clothing. Body mass was measured to the nearest 0.1 kg using a digital scale, and BMI was calculated. WC was measured 4 cm above the umbilicus at the end of a normal expiration [20]. Two measurements were taken for each variable with a third required if a difference greater than 4 mm for height and WC and 0.4 kg for weight [21, 22] existed. For height and weight, the average of the two measurements was reported, and the median value was reported if three measurements were obtained [23]. Waist-to-height ratio (WHR) was calculated as the WC divided by the height (cm). As a marker of biological maturity, each individuals' age at peak height velocity (APHV) and time from peak height velocity (TPHV) was calculated using a gender specific equation [24].

#### 2.2.2. Resting Heart Rate and Blood Pressure

 Testing sessions began with 10 min. of supine rest to ensure representative resting measurements prior to the commencement of the vascular assessment [25]. Continuous heart rate via a single-lead electrocardiograph and brachial blood pressure (BP) measurements via an automated applanation tonometer with oscillometric cuff calibration (model CBM-7000; Colin Medical Instruments, San Antonio, TX) were collected. All signals (including those described below) were acquired simultaneously using a commercially available data acquisition system (Powerlab model ML795, ADInstruments, Colorado Springs, USA) and software program (LabChart 7; ADInstruments Inc., Colorado Springs, CO, USA). At the end of the vascular assessment, four measurements of seated brachial artery pressure were obtained using an automated sphygmomanometer (Dinamap Pro 100, Critikon LCC, Tampa, FL). The first measurement was used for calibration purposes only and the average of the following three measures was reported [26].

### 2.3. Vascular Assessment

#### 2.3.1. Pulse Wave Velocity

 Baseline measurements of PWV were acquired through electrocardiography and photoplethysmography. Both central and peripheral PWV (cPWV and pPWV, resp.) were determined from 20 continuous heart cycles using the equation [13]:

(1) $$\text{PWV}=\frac{D}{\Delta t},$$

where *D* is the distance between measurement sites and Δ*t* is the pulse transit time. Arterial waveforms at the common carotid, femoral, and dorsalis pedis arteries were collected using photoplethysmograph (PPG) sensors (IR Plethysmograph; Model MLT1020PPG; ADInstruments, Colorado Springs) on the right side of the body. PPG signals were bandpass-filtered (5–30 Hz) with the lower (≤5 Hz) and higher frequencies (≥30 Hz) removed in order to assist in the detection of the foot of each waveform. The foot of each waveform was identified as the minimum value of the digitally filtered signal [27] and corresponds to the end of diastole, when the steep rise in the wave begins and appears as a sharp inflection of the original signal [28].

 Central PTT was determined using the subtraction method [29]. Similarly, cPWV path length was calculated by subtracting the surface distance between the sternal notch and the carotid PPG placement from that of the sternal notch and the femoral PPG placement. Peripheral pulse transit time was determined as the time delay between the arrival of the femoral artery pulse wave and the dorsalis pedis artery pulse wave [19], with the path length measured as the distance between these two sites. Anthropometric measuring tape was used to measure the straightline distances between skin sites (sternal notch to the placement of each PPG sensor) along the surface of the body.

#### 2.3.2. Carotid Distensibility and Intima-Media Thickness

 Direct measurements of carotid distensibility were acquired as previously described [30] using a combination of high-resolution, two-dimensional, B-mode ultrasound images (System FiVe; GE Medical Systems, Horten, Norway) and applanation tonometry (model SPT-301; Millar Instruments, Houston, TX, USA). A hand-held tonometer was positioned over the point of greatest pulsation and held in a fixed position for ten consecutive heart cycles while ultrasound images of the left common carotid artery were collected simultaneously. Absolute carotid artery systolic blood pressures were calculated by calibrating the relative values acquired using applanation tonometry to the calibrated brachial artery blood pressures acquired simultaneously [31, 32].

Ultrasound images were stored offline in Digital Image and Communications in Medicine (DICOM) format for later analysis using a semiautomated edge tracking system (AMS (Artery Measurement System) Image and Data Analysis. Tomas Gustavsson, gustav@alumni.chalmers.se) [30]. In each frame, carotid artery (minimum, mean, and maximum) lumen diameters were calculated from roughly 100 measurement markers along the vessel wall within the region of interest (ROI), for a total of 110 000 measures in a 10 heart cycle data sample. Distensibility was calculated using the equation [13]:

(2) $$\text{Distensibility}=\frac{\prod {\left(d_{\max}/2\right)}^{2}-\prod {\left(d_{\min}/2\right)}^{2}}{\prod {\left(d_{\max}/2\right)}^{2}\times \text{PP}},$$

where *d*_max⁡_ is the maximum diameter, *d*_min⁡_ is the minimum diameter, and PP is carotid pulse pressure, the change in pressure from DBP and SBP. The mean carotid diameter was calculated using the average of all diameters acquired throughout the ten heart cycles. The same software program and ultrasound images were used on the far wall of the carotid artery for measurement of the _c_IMT.

#### 2.3.3. Flow-Mediated Dilation Assessment

 The flow-mediated dilation (FMD) assessment has been shown to be the most reproducible and least variable of the techniques used to measure endothelial function in children [33]. With the participant in the supine position, the left arm was positioned (roughly 80° from the torso) and immobilized so that an optimal image of the brachial artery could be obtained in a comfortable position [34]. A sphygmomanometric cuff was placed on the forearm, below the medial epicondyle [35], and remained deflated while baseline data were collected. B-mode ultrasound images of the left brachial artery were collected through two-dimensional grayscale ultrasound imaging using a 10 MHz linear array probe (System FiVe; GE Medical Systems, Horten, Norway). A baseline longitudinal image of the brachial artery (3 consecutive cardiac cycles) was acquired by a single ultrasonographer. An intensity-weighted sample volume was attained and the gate width was therefore adjusted accordingly.

 To create the flow stimulus, the forearm cuff was instantaneously inflated to a standardized, suprasystolic pressure of 200 mmHg to ensure arterial inflow occlusion and ischemia of downstream vessels and tissue [35]. The cuff was instantaneously deflated after 5 min. of occlusion and the first 30 sec. of reactive hyperemic blood velocity signals were collected using pulsed-wave Doppler ultrasound. The forward and reverse frequency signals were processed by an external spectral analysis system (Neurovision 500 M, Multigon Ind; Yonkers NY) and an intensity-weighted calculated mean was output into a data acquisition system (Powerlab model ML795). B-mode ultrasound images of the brachial artery over three consecutive heart cycles were stored every 15 sec. from 30 sec. until 3 min. after cuff.

 End-diastolic frames were extracted from each sequence of images using a DICOM editing software program (Sante DICOM Editor 3.1.13, Santesoft, Athens, Greece). The semiautomated edge detection software program (AMS) was used to detect the vessel diameters within a specific ROI for the three end-diastolic frames at each time point. The peak dilation of the vessel was established as the single largest end-diastolic diameter (mm) measured from 30 sec. to 3 min. after cuff release. From this data, the absolute FMD (mm) and relative FMD (%FMD) were calculated as follows [36]:

(3) $$\begin{array}{cc} \text{Absolute}\;\text{FMD} & =\text{Peak}\;\text{Diameter}\;\left(\text{mm}\right)\\ & \;-\text{Baseline}\;\text{Diameter}\;\left(\text{mm}\right),\\ \text{Relative}\;\text{FMD} & =\left(\frac{\text{Absolute}\;\text{FMD}}{\text{Baseline}\;\text{Diameter}}\right)\times 100\%. \end{array}$$

The following equation was used to calculate shear rate (SR) for each participant [37]:

(4) $$\text{Shear}\;\text{Rate}=8\times \left(\frac{\text{Velocity}}{\text{Diameter}}\right),$$

where velocity represents the mean blood flow velocity of the velocity profile of the first 30 sec. after cuff release and the baseline brachial diameter (mm) is used for the internal artery diameter value. The area under the curve of the shear rate was calculated from the mean of the first point, using the trapezoid rule to obtain the area under the entire curve (GraphPad Prism version 4.00 for Windows, GraphPad Software, San Diego California USA, http://www.graphpad.com/). Relative FMD (%FMD) was normalized to the area under the entire SR curve and reported as %FMD/SR_AUC_:

(5) $$\text{Normalized}\;\text{FMD}=\left(\frac{\text{\%FMD}}{{\text{SR}}_{\text{AUC}}}\right).$$

This method of analysis provides values of absolute maximum dilation (mm), time to reach peak dilation (sec.), and a raw calculation of the SR stimulus (SR_AUC_).

### 2.4. Physical Activity

Habitual PA patterns were assessed using the Exercise Questionnaire adopted from Brunton and Bartlett, used in a longitudinal study describing exercise participation of adolescents with CP [38]. This recall questionnaire provides information regarding the frequency, duration, and intensity of PA performed in the previous week. This questionnaire, based on the “Previous Day Physical Activity Record” by Weston et al. (1997) [39], was used to assess PA in both the CP and control group.

### 2.5. Statistical Analysis

Statistical analyses were performed using SPSS Statistics, version 19.0 (SPSS, Inc., Chicago, IL). Data distribution was initially examined for normality using the Shapiro-Wilk's Test and homogeneity of variance using Levene's Test. Independent *t*-tests were used to compare group differences in all vascular indices, anthropometric measures, and levels of PA. Analyses of vascular indices were also completed with chronological age as a covariate. Statistical significance for all analyses was set at *P* ≤ 0.05.

## 3. Results

Characteristics of the study population are described in Table 1. The control and CP group were of similar age, height, weight, WC, WHR, and BMI. There were no group differences in resting seated brachial systolic blood pressure, diastolic blood pressure, mean arterial pressure, or resting supine heart rate.

Outcomes of the flow-mediated dilation (FMD) assessment are reported in Table 2. It must be noted that one participant with CP was removed from all FMD analysis due to inadequate ultrasound image quality of postocclusion data. One control subject was also identified as an outlier (via box plot and a response greater than 2 SD above the mean) and removed from the analysis. Thus, all statistical analyses of endothelial function (Table 2) were performed with an *n* = 10 in each group, with the exception of the preocclusion brachial diameters (*n* = 11) as all pre-occlusion data remained acceptable for analysis. There were no differences between groups (*P* > 0.05) in pre-occlusion brachial diameter (mm) or peak diameter (mm) reached during reactive hyperemia (Table 2). There were no differences in the SR stimulus or time taken to reach peak diameter between groups (Table 2).

There were no differences in baseline measures of carotid distensibility, _c_IMT, or baseline carotid diameter between groups (Table 3). One control subject was a significant outlier and removed from analysis of distensibility (CON, *n* = 10) and one CP subject could not be included in the analysis of _c_IMT due to insufficient clarity of the far wall IMT for proper identification (CP, *n* = 10). No differences were seen in cPWV or pPWV or PTT between groups (Table 3). One individual from the control group could not be included in the analysis due to an arrhythmia that did not permit appropriate analysis of the PWV data (CON, *n* = 10).

**Figure 1:**
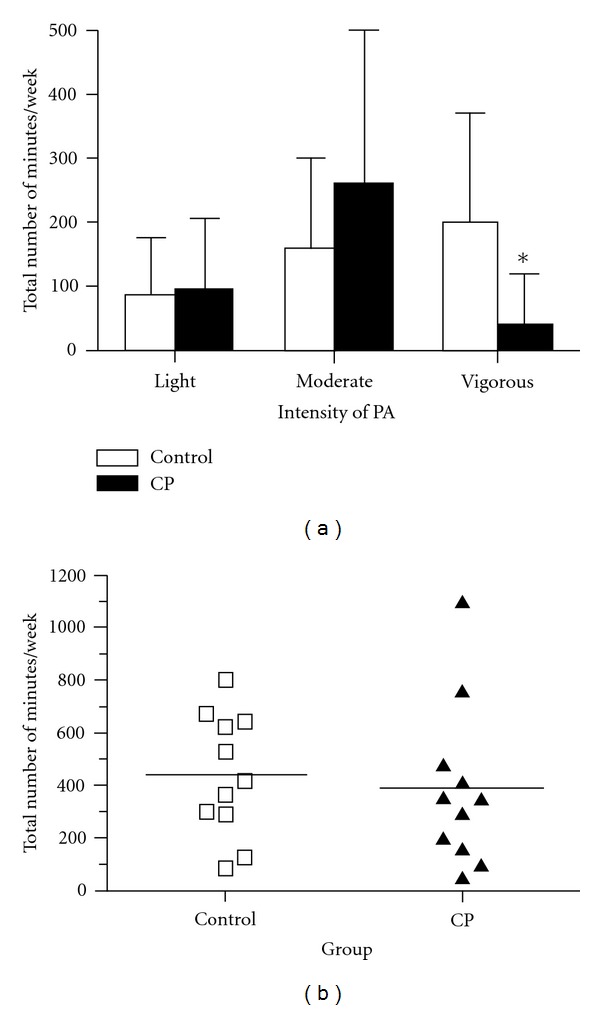
(a) Group comparisons of weekly PA according to intensity. (b) Group comparisons of total PA (summation of all three intensities) performed in one week, individual data and group means presented; *n* = 11 in each group.

The total number of minutes/week of PA in each intensity category is reported in Figure 1(a). There were no group differences in the total number of minutes spent in light and moderate PA. The CP group reported a significantly smaller amount of vigorous PA weekly than the control group (Figure 1(a)) with over 60% of individuals in the CP group reporting 0 minutes of total time spent performing vigorous PA in the previous week. Furthermore, when total PA time/week was calculated (combining each intensity of PA), there were no significant differences between groups (CP: 4260 min/week versus Controls: 4840 min/week) (Figure 1(b)).

## 4. Discussion

Over time, decreased levels of PA are generally associated with impairments of vascular function and structure and increased cardiovascular risk. This becomes particularly important when PA levels are limited in children and adolescents with a physical disability, such as cerebral palsy. Thus, early vascular assessments in this at-risk population may assist in determining potential CV risk factors. In this study we purposefully studied vascular health in the most functional adolescents with CP to contrast their PA levels and vascular health with their healthy peers. The primary findings did not confirm our hypothesis that arterial function and structure in adolescents with CP (GMFCS level I-II) are different from a healthy control group despite individuals with CP spending significantly less time performing vigorous PA in comparison to their typically developing peers.

In this study, the primary risk factor (for future cardiovascular health) of interest was level of PA, as measured using the Exercise Questionnaire [38]. Both groups spent similar amounts of time performing light-to-moderate PA; however, the CP group spent a significantly less amount of time engaging in vigorous intensity PA. Despite this discrepancy in time spent in high intensity PA, no group differences were seen in any of the measured indices of vascular health. It has been suggested that the strongest relationships between exercise interventions (comparable to levels of PA) and enhanced endothelial function exist in groups with relatively impaired FMD a priori. The tightest correlations between PA and FMD response have been shown to exist in the lowest tertiles of endothelial function [40]. Considering this, there is no reason to believe that the control group has experienced vascular dysfunction, which would predispose them to a positive vascular adaptation as a result of their higher levels of vigorous activity in comparison to the seemingly healthy CP group.

No significant differences between groups were found in cPWV or pPWV. These values were comparable to a previous study assessing PWV in a slightly younger group of healthy children (10.1 ± 0.3 yrs) who showed very similar cPWV values (4.2 ± 0.4 m/s) [14] to those in both groups in the current study (Table 3). This indicates preserved arterial stiffness at this time point for both the control and CP group. Similarities in PWV between groups in this study may be reflective of similar levels of low intensity PA, as indicated by the same amount of time spent in light and moderate intensity PA as well as the same total time spent performing PA per week (Figure 1(b)).

No differences were found between groups in either carotid distensibility or _c_IMT. Throughout the lifespan, habitual PA has been shown to positively influence arterial distensibility [14, 18]. Age-related decreases in arterial distensibility and increases in stiffness have been reported [18]; however, increased levels of PA have been suggested to delay the age-dependent loss of arterial distensibility, in proportion to the amount and/or intensity of exercise [18, 41, 42]. Although there was no difference in distensibility between the CP and control group at this time, sufficient rationale is provided for this clinical group of adolescents to increase their levels of high intensity PA at an early stage and maintain these behaviours into adulthood in an attempt to mitigate these normative age-related changes.

_c_IMT measurements were also similar between groups and were comparable to other control groups used in previous studies [43, 44]. Iannuzzi and colleagues (2004) [43] characterized the differences in _c_IMT between obese children and age-matched control subjects (6–14 years) and showed a significantly greater IMT in the obese group in comparison to the healthy controls (0.55 ± 0.08 mm versus 0.49 ± 0.09 mm). The _c_IMT of the obese children in the aforementioned study was approximately 24% and 25% greater than the _c_IMT of the present study's CP and control group, suggesting healthy vascular structure in both groups in the current study.

 In a previous study assessing the relationship between habitual PA (as measured using the double labeled water approach) and brachial FMD in 5–10-year-old children, a significant correlation was found (*r* = 0.39, *P* = 0.007), highlighting PA as the most influential variable in predicting the FMD response [9]. This group reported that physical fitness, as assessed using an incremental discontinuous treadmill-based exercise test, and levels of PA, as measured using Actigraph accelerometers, were lowest in the lowest %FMD and %FMD/SR_AUC_ tertile. These relationships between fitness, PA, and FMD response were significant, and it was concluded that PA measurements were the best predictors of endothelial (dys) function in this young group [40]. These data support the concept that PA exerts its protective effect on CV health via the endothelium and draws attention to the role of lifestyle modifications, specifically increases in levels of habitual PA in pediatric practice.

This cross-sectional study is the first to characterize indices of vascular health in higher functioning youth with CP and to make comparisons to a group of their typically developing peers. Children harbouring classic CV risk factors, including physical inactivity have been shown to exhibit impairments in vascular function and structure early in life and have an increased risk of premature atherosclerosis in adulthood [44]. It has been shown that levels of both PA and inactivity track significantly from adolescence (9 to 18 yrs) to young adulthood placing inactive children at an increased risk of becoming physically sedentary adults [45]. With evidence of physical inactivity being a significant precursor to CVD-related death and moderate levels of fitness providing protective effects against the influence of traditional risk factors on mortality [46], the value of well-established, healthy patterns of habitual PA in pediatric practice must not be overlooked. In a group of youth that may have increased susceptibility to physical inactivity, identifying any early alterations in vascular function and structure may assist in identifying preclinical vascular disease, allowing for intervention at the earliest stage possible.

## 5. Limitations

The FMD methodology used in the current study is relatively straightforward and noninvasive. However, limitations to the procedure are present. It is possible that during the FMD assessment peak dilation was underestimated as images were taken every 15 sec. for three heart cycles and not continuously for 3 min. following cuff release. This is a limitation of the storing capabilities of the equipment used; thus we chose to collect diameter data at fifteen-second intervals to attempt to represent the complete diameter profile following cuff release. The current results are limited to highly functioning, ambulatory individuals with CP and their typically developing peers. It is difficult to say if these results are applicable to prepubertal or postpubertal individuals as it can be assumed a mixed sample was represented. In this investigation, we did not control for or assess diet, vitamin ingestion or blood-borne CVD markers and therefore we cannot account for the contribution of these factors in any changes in vascular function.

 One possible explanation for our finding of similar vascular structure and function despite differences in the amount of vigorous PA is that light-to-moderate PA is the main determinant of vascular health and that vigorous exercise is not necessary to maintain normal vascular structure and function in youth. It is also possible that the method used in the current study to assess PA (CP specific questionnaire) was not sensitive enough to determine absolute differences at all intensities or that confounding factors might result in a relative underestimation of the vigorous component of exercise for youth with CP. Future studies, which include more direct measurement of activity levels, may delineate the relationship between absolute activity levels and arterial health.

## 6. Conclusion

Although no differences in vascular structure or function between youth (9–16 years) with CP and typically developing peers were observed in the current study, the establishment of techniques to assess arterial health in youth with CP is critically important for determining future CV risk in this clinical population. This study confirms the feasibility of the use of these vascular assessment techniques in this population and presents potential for future, longitudinal assessments of individuals with CP across all levels of GMFCS classification. Each measurement of cardiovascular health was well tolerated and widely accepted by both participants and their parent/guardian(s). The consequences of significantly decreased amounts of time spent in vigorous PA for youth with CP, at this time and potentially into adulthood, remain unknown. For future research it is of interest to assess whether vessel health is compromised in youth and adults with more pronounced decreased levels of PA such as those in GMFCS levels III–V. Identifying these parameters may act as a tool for risk stratification in this population, thereby permitting identification of children who would benefit most from intensified PA and/or exercise interventions.

## Figures and Tables

**Table 1:** Subject characteristics.

	Control (*n* = 11)	CP (*n* = 11)	*P* value
Age, yrs	12.4 ± 2.3	13.2 ± 2.1	0.458
Height, m	1.6 ± 0.1	1.5 ± 0.1	0.169
Weight, kg	49.3 ± 14.2	41.4 ± 8.4	0.129
APHV	13.08 ± 0.9	14.02 ± 1.3	0.062
TPHV, yrs	−0.66 ± 2.1	−0.86 ± 1.7	0.809
WC, cm	69.8 ± 8.8	67.3 ± 7.2	0.478
WHR	0.44 ± 0.05	0.45 ± 0.06	0.750
BMI, kg/m^2^	19.5 ± 3.7	18.4 ± 3.2	0.474
BMI percentile	57 ± 31	38 ± 33	0.178
Resting HR, bpm	68 ± 10	74 ± 13	0.278
Resting systolic BP, mmHg	113 ± 8	106 ± 12	0.164
Resting diastolic BP, mmHg	65 ± 5	62 ± 6	0.169
Resting MAP, mmHg	84 ± 3	81 ± 6	0.152

Values are represented as means ± SD. APHV: age at peak height velocity; TPHV: time to peak height velocity; WC: waist circumference; WHR: waist-to-height ratio; BMI: body mass index; HR: heart rate; BPM: beats per minute; BP: blood pressure; MAP: mean arterial pressure.

**Table 2:** Group comparisons of brachial vascular dimensions and FMD response.

	Control (*n* = 10)	CP (*n* = 10)	*P* value
Preocclusion diameter, mm	3.20 ± 0.37	3.08 ± 0.48	0.803
RH peak diameter, mm	3.40 ± 0.39	3.48 ± 0.38	0.815
Absolute FMD, mm	0.19 ± 0.11	0.33 ± 0.21	0.075
Relative FMD (%FMD)	6.1 ± 3.6	11.1 ± 7.8	0.080
Normalized (%FMD/SR_AUC_)	0.0027 ± 0.0015	0.0046 ± 0.0033	0.126
Mean SR	530 ± 250	544 ± 198	0.788
Time to peak, s	110 ± 45	102 ± 46	0.826
PP, mmHg	48 ± 10	45 ± 13	0.523

Values are represented as means ± SD. RH: reactive hyperemia; FMD: flow mediated dilation; SR: shear rate; SR_AUC_: shear rate area under the curve.

*Note. n* = 11 in both groups for baseline brachial diameter.

**Table 3:** Group comparisons of PTT, PWV, carotid vascular dimensions, and carotid distensibility.

	Control (*n* = 10)	CP (*n* = 11)	*P* value
Central PTT	0.103 ± 0.032	0.089 ± 0.013	0.454
cPWV (m/s)	4.1 ± 0.9	4.3 ± 0.6	0.977
Peripheral PTT	0.108 ± 0.012	0.111 ± 0.028	0.768
pPWV (m/s)	7.6 ± 1.1	7.1 ± 1.7	0.450
Baseline diameter, mm	5.73 ± 0.29	5.63 ± 0.74	0.690
_ c_IMT, mm	0.41 ± 0.03	0.42 ± 0.04	0.576
Wall/lumen ratio	0.072 ± 0.007	0.077 ± 0.012	0.832
Distensibility, mmHg^−1^	0.008 ± 0.002	0.008 ± 0.002	0.474
Compliance, mm^2^/mmHg	0.19 ± 0.03	0.17 ± 0.06	0.376
PP (mmHg)	36 ± 10	42 ± 11	0.208

Values are represented as means ± SD. PTT: pulse transit time; PWV: pulse wave velocity, distensibility and compliance: Control, *n* = 10; IMT: intima-media thickness and wall/lumen ratio: CP, *n* = 10; PP: pulse pressure.
